# Co-expression of xerophyte *Zygophyllum xanthoxylum ZxNHX* and *ZxVP1-1* confers enhanced salinity tolerance in chimeric sugar beet (*Beta vulgaris* L.)

**DOI:** 10.3389/fpls.2015.00581

**Published:** 2015-07-28

**Authors:** Guo-Qiang Wu, Rui-Jun Feng, Suo-Min Wang, Chun-Mei Wang, Ai-Ke Bao, Li Wei, Hui-Jun Yuan

**Affiliations:** ^1^School of Life Science and Engineering, Lanzhou University of Technology, Lanzhou, China; ^2^State Key Laboratory of Grassland Agro-ecosystems, College of Pastoral Agriculture Science and Technology, Lanzhou University, Lanzhou, China; ^3^Lanzhou Institute of Husbandry and Pharmaceutical Science, Chinese Academy of Agricultural Sciences, Lanzhou, China

**Keywords:** salinity tolerance, tonoplast Na^+^/H^+^ antiporter, H^+^-PPase, Na^+^ compartmentalization, sugar beet

## Abstract

Salinity is one of the major abiotic stresses that limit the growth and productivity of sugar beet (*Beta vulgaris* L.). To improve sugar beet’s salinity tolerance, the *ZxNHX* and *ZxVP1-1* genes encoding tonoplast Na^+^/H^+^ antiporter and H^+^-PPase from xerophyte *Zygophyllum xanthoxylum* were co-expressed by *Agrobacterium tumefaciens*-mediated transformation. It is showed here that co-expression of *ZxNHX* and *ZxVP1-1* confers enhanced salinity tolerance to the transformed sugar beet plants compared with the wild-type (WT) plants. The chimeric plants grew well in the presence of high salinity (400 mM NaCl), whereas WT plants displayed chlorosis and died within 8 days. Compared to WT plants, the chimeric plants co-expressing *ZxNHX* and *ZxVP1-1* accumulated more proline, Na^+^ and K^+^ in their leaves and petioles when exposed to high salinity, which caused lower solute potential, retained more water and thus subjected to lesser cell membrane damage. Interestingly, the chimeric plants accumulated higher sucrose, glucose and fructose contents in their storage roots than WT plants in the absence or presence of high salinity. Our results suggested that co-expression of *ZxNHX* and *ZxVP1-1* improved the osmoregulatory capacity in chimeric sugar beet through increased compartmentalization of ions into the vacuoles by enhancing the activity of proton pumps and thus mitigated Na^+^-toxicity for plants.

## Introduction

Salinity is a major abiotic stress limiting growth and productivity of plants in many areas of the world due to increasing use of poor quality of water for irrigation and soil salinization (Munns and Tester, 2008; Rozema and Flowers, 2008; Zhang and Shi, 2013). Saline stress to most plant species mainly accounts for the enhancement in cytoplasmic osmotic stress and Na^+^-specific toxicity (Munns and Tester, 2008; Maathuis et al., 2014). Excessive accumulation of Na^+^ in the cytosol leads to inhibitions of protein synthesis, many enzymatic reactions, and photosynthetic processes (Kronzucker et al., 2013). Plants have evolved various adaptive strategies to cope with saline stress (Shabala, 2013; Zhang and Shi, 2013; Roy et al., 2014). One of those that plant cells employ for the alleviation of excess cytosolic Na^+^ is to compartmentalize Na^+^ into the vacuoles (Yamaguchi et al., 2013). Compartmentalization of Na^+^ into vacuoles not only protects the cytoplasm from Na^+^-toxicity, but also allows plant to use Na^+^ as a benefit osmiticum for lowering its cellular osmotic potential and as such prevents water loss (Kronzucker and Britto, 2011; Shabala, 2013; Maathuis et al., 2014). It was well known that Na^+^ compartmentalization is thought to be mediated through tonoplast Na^+^/H^+^ antiporter (NHX), which are driven by proton motive forces across tonoplast generated by H^+^ transporting pumps, such as H^+^-ATPase and H^+^-PPase (VP) (Bassil and Blumwald, 2014). Theoretically, overexpression of the tonoplast Na^+^/H^+^ antiporter and H^+^-PPase genes should enhance the sequestration of Na^+^ into the vacuole, thus alleviating the toxicity of Na^+^ in the cytosol and increasing vacuolar osmoregulatory capacity, which could confer salinity tolerance to plants (Apse et al., 1999; Bhaskaran and Savithramma, 2011; Bao et al., 2014).

Indeed, over the last 16 years, numerous studies have demonstrated that overexpression of the genes encoding tonoplast Na^+^/H^+^ antiporters conferred enhanced salinity tolerance in various transgenic plants, such as *Arabidopsis thaliana* (Apse et al., 1999; Bayat et al., 2011), rice (*Oryza sativa*) (Ohta et al., 2002; Chen et al., 2007a), maize (*Zea mays*) (Chen et al., 2007b), wheat (*Triticum aestivum*) (Xue et al., 2004), tomato (*Solanum lycopersicum*) (Zhang and Blumwald, 2001; Yarra et al., 2012), rapeseed (*Brassica napus*) (Zhang et al., 2001; Rajagopal et al., 2007), soybean (*Glycine max*) (Sun et al., 2006; Li et al., 2010a), apple (*Malus* × *domestica*) (Li et al., 2010b), kiwifruit (*Actinidia deliciosa*) (Tian et al., 2011), peanut (*Arachis hypogaea*) (Banjara et al., 2012), cowpea (*Vigna unguiculata*) (Mishra et al., 2014), alfalfa (*Medicago sativa*) (Li et al., 2011; Zhang et al., 2014), and sweet potato (*Ipomoea batatas*) (Fan et al., 2014). Genetic engineering of stronger vacuolar proton pumping for improved plant salinity and/or drought resistance has also been achieved by expression of genes encoding tonoplast H^+^-PPases in transgenic plants of *Arabidopsis* (Gaxiola et al., 2001; Yao et al., 2012; Gamboa et al., 2013), alfalfa (Bao et al., 2009), creeping bentgrass (*Agrostis stolonifera*) (Li et al., 2010c), cotton (*Gossypium hirsutum*) (Pasapula et al., 2011), and peanut (Qin et al., 2013). It has been demonstrated that co-expression of *Pennisetum glaucum* tonoplast Na^+^/H^+^ antiporter *PgNHX1* and *Arabidopsis* H^+^-PPase *AVP1* genes confers higher salinity tolerance than expression of single *PgNHX1* or *AVP1* in transgenic tomato (Bhaskaran and Savithramma, 2011). Similar findings were observed in transgenic tobacco co-expressing wheat tonoplast Na^+^/H^+^ antiporter *TNHXS1* and H^+^-PPase *TVP1* genes (Gouiaa et al., 2012), and cotton co-expressing *Arabidopsis AtNHX1* and *AVP1* (Shen et al., 2015).

*Zygophyllum xanthoxylum* is a salt-accumulating xerophyte that colonizes arid areas in northwestern of China (Ma et al., 2012; Yue et al., 2012). It has been shown that *Z. xanthoxylum* can accumulate larger quantities of Na^+^ than K^+^ in shoot tissues for osmotic adjustment even at low salt soils (Wang et al., 2004). Further studies showed that low concentrations of Na^+^ significantly increased growth and alleviated water stress in this species (Ma et al., 2012; Yue et al., 2012). The *ZxNHX* and *ZxVP1-1* genes, encoding tonoplast Na^+^/H^+^ antiporter and H^+^-PPase, were cloned from *Z. xanthoxylum* (Wu et al., 2011). A positive correlation was observed between up-regulation of *ZxNHX* and accumulation of Na^+^ in leaf of *Z. xanthoxylum* in the presence of salt, suggesting that ZxNHX is involved in compartmentalization of Na^+^ in shoots (Wu et al., 2011). Our recent studies showed that *ZxNHX* controls Na^+^ and K^+^ homeostasis at the whole-plant level in *Z. xanthoxylum* by feedback regulation of the expression of genes involved in their transport (Yuan et al., 2015). Bao et al. (2014) reported that coordinating expression of *ZxNHX* and *ZxVP1-1* enhanced salt- and drought-tolerance in transgenic *Lotus corniculatus* by increasing Na^+^, K^+^, and Ca^2+^ accumulation. These results suggested that coordinating expression of these two genes is more valuable way for obtaining transgenic plants with enhanced stress tolerance. However, there are no reports regarding improving salinity tolerance of sugar crops by co-expressing tonoplast Na^+^/H^+^ antiporter and H^+^-PPase.

Sugar beet (*Beta vulgaris* L.), a species of Chenopodiaceae family, is an important sugar crop that supplies approximately 35% of the world’s sugar (Liu et al., 2008). Sugar beet is regarded as one of the relatively more salinity-tolerant crops (Wu et al., 2013, 2015a); therefore, it is a good choice for the reclamation of saline land. However, sugar beet’s growth and development, especially its yield and sugar quality, are negatively affected by high salinity. Therefore, breeding sugar beet varieties with higher salinity tolerance is necessary for this important crop to adapt to high salinity. A consider amount of time is required to select salinity tolerance plants through traditional breeding procedure. The genetic engineering approach, however, provides an efficient way to improve salinity tolerance in sugar beet. Here we investigated the possibility of co-expression of *Z. xanthoxylum ZxNHX* and *ZxVP1-1* to enhance salinity tolerance in sugar beet.

## Materials and Methods

### Plasmid and Bacteria Strains

The plasmid pCAMBIA1302-*ZxNHX*-*ZxVP1*-*1*, which contains the cauliflower mosaic virus 35S (CaMV 35S) promoter driving *ZxNHX* (GenBank accession number: EU103624, encoding tonoplast Na^+^/H^+^ antiporter) and *ZxVP1*-*1* (GenBank accession number: EU103625, encoding tonoplast H^+^-PPase) from *Z. xanthoxylum*, has been previously constructed in our laboratory (Bao et al., 2014). The constructs were mobilized into *Agrobacterium tumefaciens* strain GV3101 by electroporation for subsequent plant transformation (Bao et al., 2014). *Agrobacterium* suspension was obtained after incubation in 100 mL Luria-bertani (LB) liquid medium (pH 7.0) containing 50 μg mL^–1^ kanamycin, 50 μg mL^–1^ gentamicin, and 25 μg mL^–1^ rifampicin overnight at 28°C under constant rotation at 200 rpm and resuspension in the appropriate volume of free-antibiotics LB liquid medium to an OD_600_ of 0.6–0.8.

### Plant Materials and Transformation

Seeds of sugar beet (*B. vulgaris* L. cv. “Gantang7”) were kindly provided by Wuwei Sannong Seed Technology Co., Ltd., Gansu province, China, in mid March 2013. Seeds were surface sterilized for 1 min in 75% ethanol (*v*/*v*) and rinsed 3 times with distilled water, soaked in distilled water for 1 day and then germinated at 25 °C in the dark for 3 days. Uniform seedlings were carefully transferred to plastic containers (5 cm × 5 cm × 5 cm; two seedlings/container) filled with vermiculite and irrigated with the modified Hoagland nutrient solution containing 2.5 mM KNO_3_, 1 mM NH_4_H_2_PO_4_, 0.5 mM Ca(NO_3_)_2_, 0.5 mM MgSO_4_, 60 μM Fe-Citrate, 92 μM H_3_BO_3_, 18 μM MnCl_2_·4H_2_O, 1.6 μM ZnSO_4_·7H_2_O, 0.6 μM CuSO_4_·5H_2_O, and 0.7 μM (NH_4_)_6_Mo_7_O_24_·4H_2_O. Solutions were renewed every 3 days. The seedlings were grown in a greenhouse, where the temperature was regulated 20°C at night and 25°C in the day and the relative humidity averaged 65 and 75% for day and night, respectively. The daily photoperiod was 16/8 h (day/night) and the light flux density during the light period was between 550 and 600 μmol m^–2^ s^–1^. 2-week-old seedlings were used for genetic transformation.

The transformation was performed using the procedure as described by Weeks et al. (2008) with a slight modification. Briefly, after the cotyledons of 2-week-old seedlings expanded and the apical node emerged, the apical node was excised and the wound covered with cotton wool wetted with *Agrobacterium* suspension as described above. For wild-type (WT) control, the *Agrobacterium* suspension was replaced by distilled water. The seedlings were put in the dark for 3 h and then cotton was removed. Thereafter, the seedlings were transferred into and grown in the greenhouse and irrigated with modified Hoagland nutrient solution as described above. 5 days later, a new apical node emerged from the wound region and formed new shoots 25 days later. All the T0 transformed plants were used for molecular characterization.

### Molecular Characterization of Chimeric Sugar Beet

For PCR analysis, plant genomic DNA was extracted from fresh leaves of putative chimeric and WT plants using the Ezup Column Genome DNA Isolation Kit (Sangon, Shanghai, China). The *ZxNHX* and *ZxVP1-1* genes in chimeric and WT plants were amplified from same DNA templates using the following sets of specific primers: (i) 5′-CATCGGTGGTGCTTTTCAAT-3′ and 5′-GCAGCTCTACCAGCCATCAC-3′ for *ZxNHX*, (ii) 5′-GCTGGAATCGAATTTGTGGA-3′ and 5′-TGCAGCCTTATGTGCATCTG-3′ for *ZxVP1-1*. The PCR amplification conditions for the *ZxNHX* and *ZxVP1-1* fragments were carried out with initial denaturation at 94 °C for 2 min, followed by 30 cycles of 94°C for 30 s, 54°C for 45 s, 72°C for 30 s, and final extension at 72°C for 10 min. PCR products were electrophoresed on a 1.2% agarose gel, respectively.

For RT-PCR analysis, total RNA was extracted from 50 mg of young leaves of PCR-positive plants and WT plants with Trizol reagent (Sangon, Shanghai, China) according to manufacturer’s instructions. DNase treated total RNA samples were used for the synthesis of first strand cDNA using M-MLV Reverse Transcriptase Kit (Sangon, Shanghai, China). PCR amplification of the *ZxNHX* and *ZxVP1-1* gene was carried out according to the conditions described above and *ACTIN* was used as a internal control in the semi-quantitative RT-PCR. The primer sequences of *ACTIN* are 5′-GTGGTCGTAC AAC(A/T)GGTATTGTG-3′ and 5′-GA(A/G/T)CCTCCAATCCAGACACTG-3′, which give a 598 bp fragment with cDNA. The all amplified products were electrophoresed on 1.2 % agarose gel, and visualized by AlphaImager (ver. 4.0.1; Alpha Innotech Co., San Leandro, CA, USA) for subsequent analysis, respectively. Experiments were repeated at least three times.

### Treatments of Chimeric and Wild-type Plants

Uniform T0 chimeric (uniform expression levels of genes) and WT plants were exposed to the modified Hoagland nutrient solution as described above supplemented without or with 400 mM NaCl for 8 days, respectively. Treatment solution was renewed every 2 days. All the plants were grown in the greenhouse as described above.

### Determination of Plant Growth and Water Content

At the end of treatments, the plants were divided into separate leaf, petiole, storage root, and lateral root fractions. Fresh weights (FW) of the tissues were weighed immediately. The samples were then dried in oven at 80°C for 72 h to determine dry weights (DW). Water content (WC) was calculated according to the following equation as described by Wu et al. (2013): WC = (FW – DW)/DW.

### Determination of Malondialdehyde (MDA) Concentration

Malondialdehyde (MDA) was determined using the thiobarbituric acid (TBA) protocol as described by Peever and Higgins (1989) with slight modifications. The absorbances at 450, 532, and 600 nm (A_450_, A_532_, and A_600_, respectively) were measured using an UV-VIS spectrophotometer (UV-300 OPC, Mapada Co., Shanghai, China). The concentration of MDA in nmol/g FW was calculated according to the equation of Bao et al. (2009): MDA concentration (nmol/g FW) = *C* (μmol/L) × *V* (L)/FW (g) × 1000, where *C* (μmol/L) = 6.45 × (A_532_ – A_600_) – 0.56 A_450_, and *V* represents the volume (L) of extracting solution.

### Determination of Solute Potential (Ψ_*S*_)

Plant tissues (leaf, petiole, storage root, and lateral root) were frozen in liquid nitrogen and then stored at –80°C until the analysis of solute potential (Ψ_*s*_). Ψ_*s*_ was detected with the sap squeezed out from the thawed shoot at 25°C with a cryoscopic osmometer (Osmomat-030, Gonotec GmbH, Berlin, Germany). The reading (mmol kg^–1^) was used to calculate the Ψ_*s*_ in MPa with the formula Ψ_*s*_ = -moles of solute × R × T, where R = 0.008314 and T = 298.8 (Ma et al., 2012).

### Determination of Proline and Sugar Contents

Proline was measured according to the method of Bates et al. (1973) using the ninhydrin reagent. Proline content was estimated using an UV-VIS spectrophotometer at 520 nm and determined from calibration curve using L-Proline (Sangon, Shanghai, China) as standard. Sugars contents were determined according to the method as described by Liu et al. (2008). Briefly, sugars were extracted from dried plant tissues in 80% ethanol at 80°C for 40 min. Sucrose, glucose, and fructose contents were read at 480, 630, and 480 nm and determined from calibration curve using Sucrose, Glucose and D-Fructose (Sangon, Shanghai, China) as standard, respectively.

### Measurement of Na^+^, K^+^, and Ca^2+^ Concentrations

Na^+^, K^+^, and Ca^2+^ concentrations were detected according to the method described by Wu et al. (2013, 2015a). Briefly, Na^+^, K^+^, and Ca^2+^ were extracted from dried plant tissues (leaf, petiole, storage root, and lateral root) in 100 mM acetic acid at 90°C for 2 h and ions analysis was performed by a flame spectrophotometer (2655-00, Cole-Parmer Instrument Co., Vernon Hills, USA) (Wu et al., 2013, 2015a).

### Statistical Analysis

Data were performed by one-way analysis of variance (ANOVA) using statistical software (SPSS 19.0, Chicago, USA). Duncan’s multiple range tests were used to detect significant difference between means at a significant level of *P* < 0.05.

## Results

### Production and Molecular Characterization of Chimeric Sugar Beet Plants Co-expressing *ZxNHX* and *ZxVP1-1*

**FIGURE 1:**
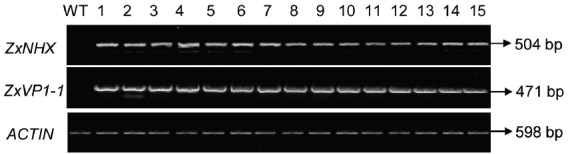
**RT-PCR analysis of ***ZxNHX*** and ***ZxVP1-1*** genes in T0 chimeric sugar beet plants.** Specific PCR products of *ZxNHX* for 504 bp and *ZxVP1-1* for 471 bp were detected in the new leaves of chimeric sugar beet plants, respectively. WT, wild type plant; 1–15, the T0 chimeric plants of co-expressing *ZxNHX* and *ZxVP1-1*. A 598 bp *ACTIN* fragment was amplified by RT-PCR as an internal control.

The 35S-*ZxNHX1-ZxVP1-1* construct described by Bao et al. (2014) was introduced into sugar beet via *Agrobacterium*-mediated transformation (Weeks et al., 2008). To validate whether the *ZxNHX* and *ZxVP1-1* genes from xerophyte *Z. xanthoxylum* integrated into sugar beet genome, PCR reactions were performed with specific primers to amplify the fragments of *ZxNHX* and *ZxVP1-1*, respectively. We totally obtained 40 T0 chimeric plants that showed the fragments of 504 bp for *ZxNHX* and 471 bp for *ZxVP1-1* by PCR amplification, respectively. A stable transformation efficiency of 9.95% was observed using a total of 402 plants in three different experiments generating 40 T0 chimeric sugar beet plants. The expression levels of *ZxNHX* and *ZxVP1-1* were monitored by RT-PCR performed on young leaves of the all T0 chimeric plants and WT plants, respectively. As expected, co-expression of *ZxNHX* and *ZxVP1-1* in all PCR-positive T0 chimeric plants were showed and their expression levels almost were consistent among all different T0 chimeric plants, while the WT plants did not show amplification fragments (Figure 1). Sugar beet plants were difficultly propagated from their leaves and petioles, therefore, 36 of these T0 chimeric plants were directly chose to further analyze physiological parameters.

### Co-expression of *ZxNHX* and *ZxVP1-1* Enhances Salinity Tolerance in Chimeric Sugar Beet

**FIGURE 2:**
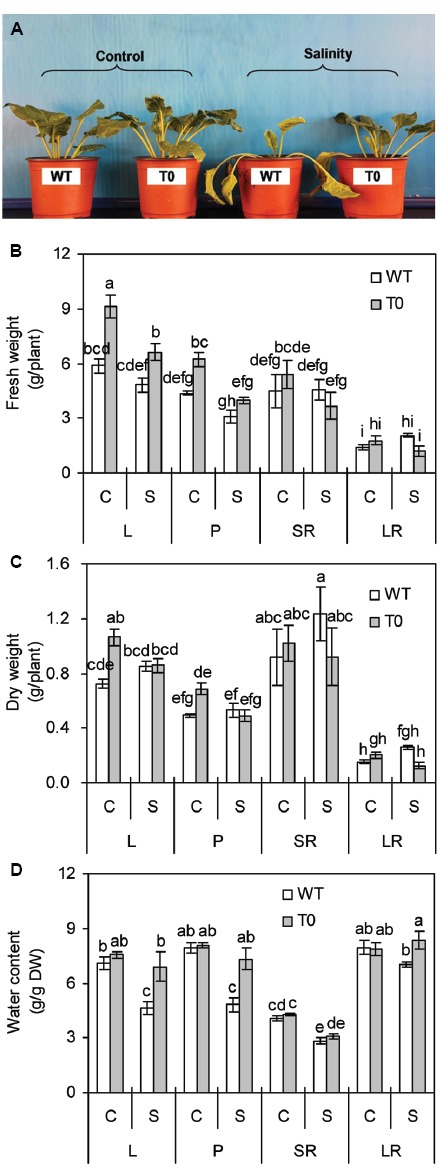
**Morphology (A), fresh weight (B), dry weight (C), and water content (D) of 2-month-old wild-type (WT) and chimeric plants (T0) co-expressing ***ZxNHX*** and ***ZxVP1-1*** grown under control (C) and salinity (S) condition (treated with 400 mM NaCl for 8 days).** L: leaf; P: petiole; SR: storage root; LR: lateral root. Data in **(B–D)** are mean ± SE (*n* = 6) and bars indicate the SE. For T0 chimeric plants, values are derived from a combination of single plants each from one of six lines. Columns with different letters indicate significant difference at *P* < 0.05 (Duncan’s test).

Both T0 chimeric and WT plants grew well vigorously under control condition, while chimeric plants developed faster to larger size than WT plants. When exposed to high salinity (400 mM NaCl), WT plants wilted and died, but the chimeric plants grew well (Figure 2A). These results indicated that co-expression of *ZxNHX* and *ZxVP1-1* enhanced salinity tolerance in chimeric sugar beet plants.

It was observed that FW and dry weight (DW) in leaf and petiole of chimeric plants were significantly higher than that of WT plants under control condition (*P* < 0.05) (Figures 2B,C). When subjected with salinity, FW in leaf of chimeric plants was 38% higher than that of WT plants. However, no significant differences in tissues DW were found between WT and chimeric plants (Figure 2C). Salinity caused a significant reduction of WC in storage root of both WT and chimeric plants (*P* < 0.05). WC of leaf and petiole in WT plants significantly reduced by 35 and 64% under saline stress, respectively, whilst remained unchanged in chimeric plants (Figure 2D). WC in leaf, petiole, and lateral root of chimeric plants was 49, 51, and 19% higher than that in WT plants under saline stress, respectively (*P* < 0.05) (Figure 2D). These results suggested that chimeric sugar beet plants co-expressing *ZxNHX* and *ZxVP1-1* displayed greater water retention capacity compared with WT plants.

### Co-expression of *ZxNHX* and *ZxVP1-1* Maintains the Stability of Cell Membrane in Chimeric Sugar Beet Under Saline Stress

**FIGURE 3:**
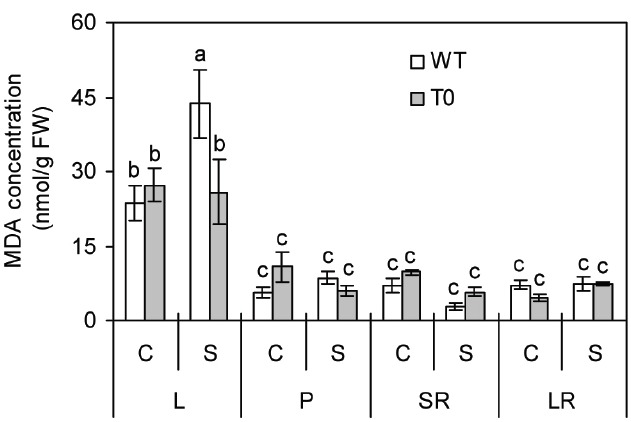
**Malondialdehyde (MDA) concentration in leaf (L), petiole (P), storage root (SR), and lateral root (LR) of 2-month-old wild-type (WT) and chimeric plants (T0) co-expressing ***ZxNHX*** and ***ZxVP1-1*** grown under control (C) and salinity (S) condition (treated with 400 mM NaCl for 8 days).** Data are mean ± SE (*n* = 6) and bars indicate the SE. For T0 chimeric plants, values are derived from a combination of single plants each from one of six lines. Columns with different letters indicate significant difference at *P* < 0.05 (Duncan’s test).

To investigate the stability of membrane in WT and chimeric plants under saline stress, MDA concentration that represents the degree of cell membrane damage was determined (Yazici et al., 2007; Han et al., 2014). Under control condition, no significant differences were seen in all the tissues from WT and chimeric plants. When subjected to saline stress, MDA concentration in leaf was 69% lower in chimeric plants than in WT plants (*P* < 0.05) (Figure 3). These results suggested that the cell membrane of chimeric sugar beet was healthier or subjected to less damage than WT plants under saline stress.

### Chimeric Sugar Beet Plants Retain More Solute in Shoot

**FIGURE 4:**
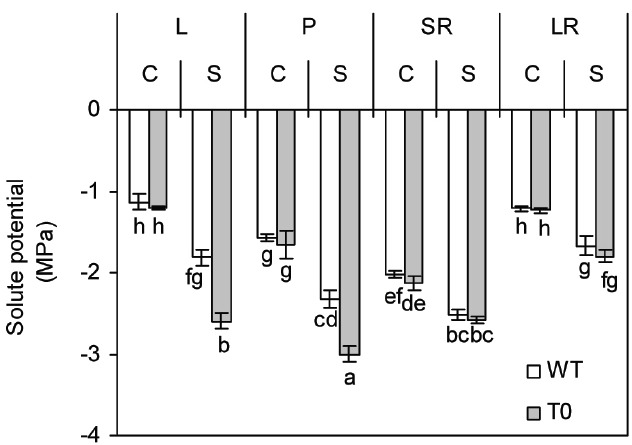
**Solute potential in leaf (L), petiole (P), storage root (SR), and lateral root (LR) of 2-month-old wild-type (WT) and chimeric plants (T0) co-expressing ***ZxNHX*** and ***ZxVP1-1*** grown under control (C) and salinity (S) condition (treated with 400 mM NaCl for 8 days).** Data are mean ± SE (*n* = 6) and bars indicate the SE. For T0 chimeric plants, values are derived from a combination of single plants each from one of six lines. Columns with different letters indicate significant difference at *P* < 0.05 (Duncan’s test).

To assay the amount of solute in the tissues, the tissues solute potential (*Ψ*_*s*_) of WT and chimeric plants was determined. Under control condition, the *Ψ*_*s*_ of storage root was significantly lower than that of other tissues in either WT or chimeric plants (*P* < 0.05). When exposed to saline stress, all the tissues *Ψ*_*s*_ of both chimeric and WT plants dropped significantly (*P* < 0.05). However, the *Ψ*_*s*_ of both leaf and petiole was more negative in chimeric plants than that in WT plants (*P* < 0.05) (Figure 4). These results suggested that chimeric plants had higher osmoregulatory capacity compared to WT plants.

**FIGURE 5:**
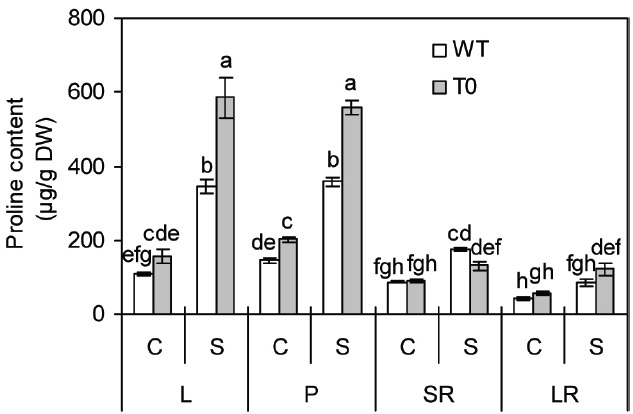
**Proline content in leaf (L), petiole (P), storage root (SR), and lateral root (LR) of 2-month-old wild-type (WT) and chimeric plants (T0) co-expressing ***ZxNHX*** and ***ZxVP1-1*** grown under control (C) and salinity (S) condition (treated with 400 mM NaCl for 8 days).** Data are mean ± SE (*n* = 6) and bars indicate the SE. For T0 chimeric plants, values are derived from a combination of single plants each from one of six lines. Columns with different letters indicate significant difference at *P* < 0.05 (Duncan’s test).

In the absence of salinity, proline content in petiole of chimeric plants was 38% higher than that of WT plants, however, in other tissues, no clear differences were seen between WT and chimeric plants (Figure 5). In the presence of salinity, proline content in shoots (leaf and petiole) of both chimeric plants and WT plants remarkably increased, whereas to a lesser degree in WT than in chimeric plants. It was also observed that chimeric plants accumulated more proline in their leaf and petiole by 70% and 55% compared with WT plants, respectively (*P* < 0.05) (Figure 5).

### Chimeric Sugar Beet Plants Accumulate More Na^+^, K^+^ in Shoots and More Ca^2+^ in Lateral Root

**FIGURE 6:**
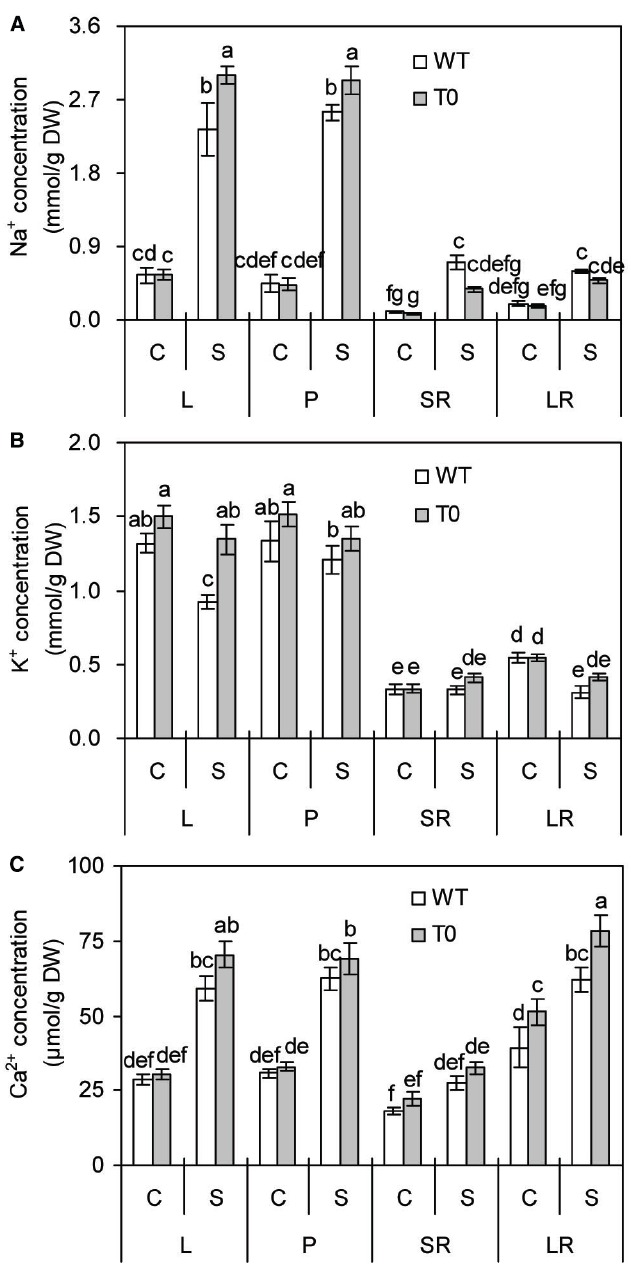
**Na^+^ (A), K^+^ (B), and Ca^2+^ (C) concentrations in leaf (L), petiole (P), storage root (SR), and lateral root (LR) of 2-month-old wild-type (WT) and chimeric plants (T0) co-expressing ***ZxNHX*** and ***ZxVP1-1*** grown under control (C) and salinity (S) condition (treated with 400 mM NaCl for 8 days).** Data are mean ± SE (*n* = 6) and bars indicate the SE. For T0 chimeric plants, values are derived from a combination of single plants each from one of six lines. Columns with different letters indicate significant difference at *P* < 0.05 (Duncan’s test).

To investigate whether chimeric plants co-expressing *ZxNHX* and *ZxVP1-1* could accumulate more ions, the concentrations of Na^+^, K^+^, and Ca^2+^ in chimeric and WT plants were measured. In the absence of salinity, no remarkable differences were found in leaf, petiole, storage root and lateral root Na^+^ concentration from WT and chimeric plants. When exposed to saline stress, Na^+^ concentration of leaf and petiole increased obviously in all plants (*P* < 0.05) (Figure 6A). However, leaf and petiole Na^+^ concentrations in chimeric plants were 28 and 15% higher than those in WT plants, respectively (*P* < 0.05) (Figure 6A). Compared to control, saline stress significantly increased Na^+^ concentrations of storage root and lateral root by 3.9- and 1.7-fold in WT plants (*P* < 0.05), respectively, but not in chimeric plants. These results suggested that chimeric sugar beet accumulated more Na^+^ in shoots but less in roots.

It is clear that salinity caused a significant reduction of leaf and lateral K^+^ concentrations by 30 and 43% in WT plants compared to control (*P* < 0.05), respectively. By contrast, chimeric plants maintained stable K^+^ concentration in all the tissues under saline stress (Figure 6B). It was observed that there are no significant differences between WT and chimeric plants under control condition. However, K^+^ concentration in leaf of chimeric plants was 45% higher than that of WT plants under saline condition (*P* < 0.05). It was found that saline stress remarkably increased leaf, petiole, lateral root Ca^2+^ concentrations in both WT and chimeric plants (*P* < 0.05), whereas to a greater degree in chimeric plants than in WT plants (Figure 6C). Especially, lateral root Ca^2+^ concentration of chimeric plants was 30 or 26% higher than that of WT plants in the absence or presence of salinity.

### Chimeric Sugar Beet Plants Accumulate More Soluble Sugars in Storage Root

**FIGURE 7:**
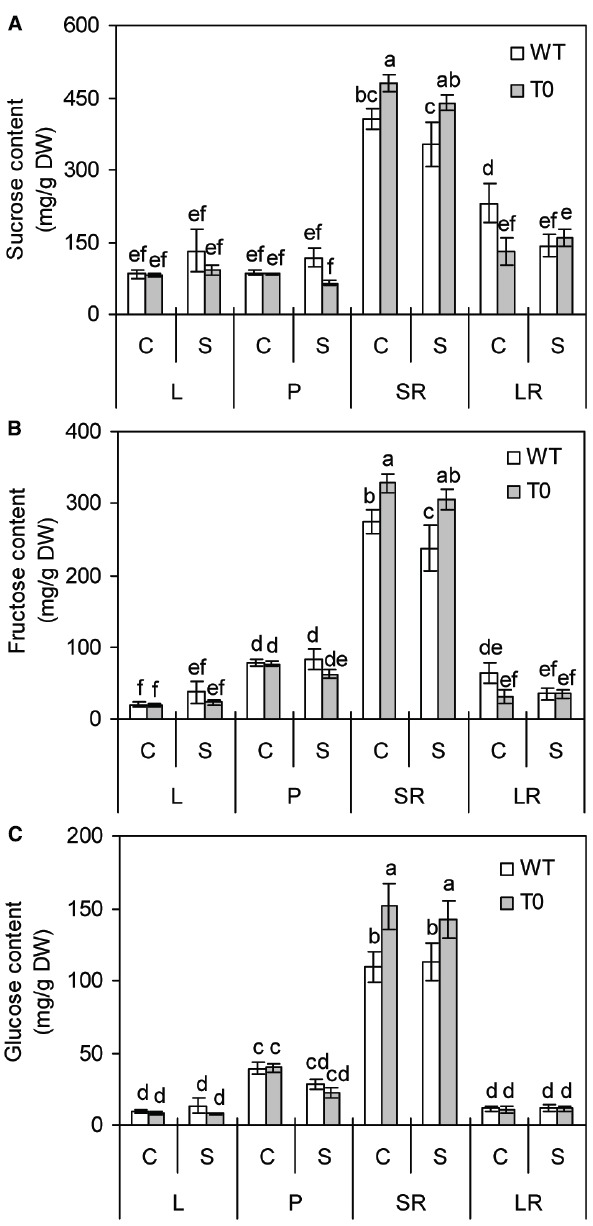
**Sucrose (A), fructose (B), and glucose (C) contents in leaf (L), petiole (P), storage root (SR), and lateral root (LR) of 2-month-old wild-type (WT) and chimeric plants (T0) co-expressing ***ZxNHX*** and ***ZxVP1-1*** grown under control (C) and salinity (S) condition (treated with 400 mM NaCl for 8 days).** Data are mean ± SE (*n* = 6) and bars indicate the SE. For T0 chimeric plants, values are derived from a combination of single plants each from one of six lines. Columns with different letters indicate significant difference at *P* < 0.05 (Duncan’s test).

To understand whether co-expression of *ZxNHX1* and *ZxVP1-1* would affect sugar metabolism in chimeric plants, we determined the sucrose, fructose and glucose contents of WT and chimeric plants grown under saline condition. It was showed that the sucrose, fructose, and glucose contents in storage root of chimeric plants were significantly higher than those of WT plants under either control or saline condition, respectively (*P* < 0.05) (Figures 7A–C). However, in other tissues, no significant differences were found between WT and chimeric plants (Figures 7A–C). It was also found that storage root displayed the highest sugars contents among all the tissues in either WT or chimeric plants (*P* < 0.05) (Figures 7A–C). These results suggested that chimeric plants can accumulate more soluble sugars in storage root.

## Discussion

In the present study, the *ZxNHX* and *ZxVP1-1* genes from xerophyte *Z. xanthoxylum* were introduced into the important sugar crop sugar beet. We obtained T0 chimeric sugar beet plants co-expressing *ZxNHX* and *ZxVP1-1* (Figure 1). However, the transgenes are present and detectable in the new upper leaves but would not be present in the non-transformed root tissues—i.e., the storage root and the lateral root would not be transgenic in the T0 plants. Thus, it is clear that these T0 plants are likely to be “chimeric” transgenic plants.

Compared with the WT plants, the T0 chimeric sugar beet plants displayed improved salinity tolerance. After stress with high salinity (400 mM NaCl), the WT plants displayed growth inhibition, chlorosis, and even death, whereas the T0 chimeric plants exhibited normal growth and survival (Figure 2A). This can be attributed to the enhanced compartmentalization of Na^+^ into vacuoles resulting from co-expression of tonoplast Na^+^/H^+^ antiporter and H^+^-PPase (Gaxiola et al., 2001, 2007; Bao et al., 2009; Yamaguchi et al., 2013; Fan et al., 2014). This mechanism could provide better protection for plant cells via mitigating the toxic effects of Na^+^ in the cytosol, maintaining ions homeostasis, and increasing vacuolar osmoregulatory capacity (Gaxiola et al., 2007; Bao et al., 2014). This notion is further supported in the present study by the measurements of ions accumulation; the chimeric plants accumulated more Na^+^ and K^+^ in their leaf and petiole as well as more Ca^2+^ in lateral root compared with the WT plants (*P* < 0.05) (Figures 6A–C). These results corroborate earlier findings of salinity tolerance by co-expressing *ZxNHX* and *ZxVP1-1* in *L. corniculatus* (Bao et al., 2014).

It was also observed that chimeric plants grew better than WT plants even under control conditions (Figures 2A–C). Similar phenotypes were observed in transgenic cotton overexpressing *AVP1* (Pasapula et al., 2011) and *L. corniculatus* co-expressing *ZxNHX* and *ZxVP1-1* (Bao et al., 2014). It was well demonstrated that tonoplast Na^+^/H^+^ antiporter and H^+^-PPase played essential roles in plant growth and development (Apse et al., 2003; Li et al., 2005; Gaxiola et al., 2007; Bassil et al., 2011). Apse et al. (2003) found that tonoplast Na^+^/H^+^ antiporters control cell expansion and leaf development by regulating vacuolar pH and ions homeostasis. These findings ware further confirmed by Bassil et al. (2011), who reported that *Arabidopsis* double mutant *nhx1*/*nhx2* exhibited remarkably reduced growth, smaller cells, shorter hypocotyls in etiolated seedlings and abnormal stamens in mature flowers compared with WT plants. Tonoplast H^+^-PPase has been shown to play an important role in facilitating auxin transport and distribution, and thus regulating auxin-related developmental processes (Li et al., 2005). The over-expression of *AVP1* resulted in increased cell division at the onset of organ formation and hyperplasia by enhancing auxin transport in transgenic *Arabidopsis*, while *avp1-1* null mutants showed severely disrupted root and shoot development by reducing auxin transport (Li et al., 2005; Gaxiola et al., 2007).

K^+^ is the major ionic osmoticum in plant cells and occurs in two major pools, in the vacuole and in the cytosol (Barragán et al., 2012). In the present study, the chimeric plants co-expressing *ZxNHX* and *ZxVP1-1* accumulated a markedly higher level of K^+^ in their leaves under saline conditions as compared with WT plants (Figure 6B). Significant enhancement in K^+^ during salinity stress has also been reported in transgenic tomato overexpressing *AtNHX1* (Leidi et al., 2010), *L. corniculatus* co-expressing *ZxNHX* and *ZxVP1-1* (Bao et al., 2014), and alfalfa overexpressing *TaNHX2* from wheat (Zhang et al., 2015). The increase in K^+^ accumulation of transgenic plants is likely to be due to increased NHX activity as these transporters are also known to use K^+^ as a substrate (Shabala and Pottosin, 2014). It was showed that the overexpression of tonoplast Na^+^/H^+^ antiporters led to more vacuolar K^+^ accumulation and greater K^+^ uptake in transgenic plants compared to WT plants (Leidi et al., 2010; Zhang et al., 2015), whereas *Arabidopsis* double mutant *nhx1/nhx2* displayed lower K^+^ contents than WT plants (Bassil et al., 2011; Barragán et al., 2012). These results implied that tonoplast Na^+^/H^+^ antiporters are crucial for the accrual of K^+^ in shoot tissues of plants.

There were evidences that accumulating more ions such as Na^+^ and K^+^ resulting from the overexpression of the genes encoding tonoplast Na^+^/H^+^ antiporter and H^+^-PPase can improve the osmoregulation capacity, which serves as a force to drive water uptake and thus, maintain the turgor of transgenic plants under salinity condition (Bao et al., 2009, 2014; Gouiaa et al., 2012). Our results supported this point again. Owing to lower leaf and petiole solute potential in chimeric sugar beet (Figure 4), leaves and petioles of chimeric plants retained more water compared with WT plants (Figure 2D). Similarly, lower solute potential was also observed in transgenic *L. corniculatus* co-expressing *ZxNHX* and *ZxVP1-1* that is in agreement with the findings of the present study and may be due to superior osmotic adjustment of transgenic plants (Bao et al., 2014).

Salinity induces oxidative stress in cells, leading to damage to cellular machinery including free-radical-mediated lipid peroxidation (Mishra et al., 2014). MDA acts as an effective indicator for measuring the degree of lipid peroxidation (Yazici et al., 2007). Under salinity condition, chimeric sugar beet plants exhibited a lower concentration of MDA in their leaf than the WT plants (Figure 3), indicative of the occurrence of less oxidative damage with co-expression of *ZxNHX* and *ZxVP1-1*. Tian et al. (2011) also reported that transgenic kiwifruit plants overexpressing *AtNHX1* showed a significantly lower MDA content than WT plants when exposed to salinity conditions. These were in accordance with results for transgenic cowpea overexpressing *VrNHX1* from mungbean (*Vigna radiata*) (Mishra et al., 2014) and sweet potato overexpressing *AtNHX1* (Fan et al., 2014). These findings perhaps ascribed to the enhanced sequestration of Na^+^ into the vacuoles, which may protect the cells from the toxicity of excess Na^+^. Therefore, it can be assumed that co-expression of *ZxNHX* and *ZxVP1-1* might protect the cell membrane from injuries induced by saline stress.

The accumulation of proline in response to high salinity has been well documented (Juan et al., 2005; Wu et al., 2015b). Proline, an important osmoprotectants, contributes to osmotic adjustment, stabilization of proteins and protein complexes, protection of sub-cellular structures like membranes and macromolecules as well as photosynthetic apparatus under salinity stress, and acts as a reactive-oxygen-species (ROS) scavenger (Mishra et al., 2014; Wu et al., 2015a). Evidence supporting the role of proline during saline stress was obtained based on salinity tolerance in transgenic tobacco plants with increased levels of proline biosynthesis (Kishor et al., 1995) and salinity tolerance of *Arabidopsis* with suppressed levels of proline degradation (Nanjo et al., 1999). In the present study, the accumulation of proline under saline stress was dramatically increased in leaf and petiole of both chimeric and WT plants. However, the accumulation was more pronounced in chimeric plants than in WT plants (Figure 5). An increase of proline content during saline stress has also been observed in *AtNHX1*-expressing transgenic sweet potato (Fan et al., 2014) and *VrNHX1*-expressing transgenic cowpea (Mishra et al., 2014). These results implied that the sequestration of excessive Na^+^ into the vacuoles caused by increasing tonoplast Na^+^/H^+^ transport activity may activate the expression of salinity tolerance-related genes, which contribute to the enhancement of proline accumulation.

It is well known that the sugar content of storage root is the main qualitative determinant of the crop, which is one of the most important raw material sources for the production of manufactured sugar throughout the world (Liu et al., 2008). Therefore, the sugar content is also an important objective in sugar beet breeding. Under either control or salinity conditions, contents of sucrose, glucose and fructose in storage root of chimeric plants were significantly higher than those in WT plants (Figures 7A–C). Similar results were observed in transgenic sugar beet overexpressing *AtNHX3* as described by Liu et al. (2008), who reported that the maintenance of ions homeostasis diminished the toxic effects of Na^+^ on the expression of enzymes associated with sucrose synthesis, allowing increased sucrose production in transgenic plants overexpressing *AtNHX3* (Liu et al., 2008). However, when grown under control or salinity conditions, no significant difference was seen on the sugar contents of shoots in WT and transgenic plants (Figures 7A–C). Recently, Pizzio et al. (2015) showed that AVP1 has essential functions independent of phloem sucrose loading in *Arabidopsis*. These are probably related to AVP1 in the vacuolar and prevacuolar compartments mediating PPi hydrolysis and lumen acidification (Pizzio et al., 2015). In addition, although the accumulations of soluble sugars in many species have been widely reported as a response to salinity stress (Ashraf and Harris, 2004; Juan et al., 2005; Nemati et al., 2011), other studies do not support this response (Ashraf, 1997). In the present study, contents of sugar in sugar beet plants were not remarkably affected by salinity stress (Figures 7A–C). Similar results were reported in this species (Wu et al., 2013, 2015a) and other plant species (Morgan, 1992). This could be attributed to the excessive accumulation of Na^+^ in shoot tissues plants that inhibits enzymes involved in carbohydrate metabolism under high salinity conditions (Munns and Tester, 2008).

In conclusion, our results demonstrated that co-expression of *ZxNHX* and *ZxVP1-1* can enhance salinity tolerance in chimeric sugar beet. It was noteworthy that chimeric plants accumulated more sucrose, glucose and fructose in their storage roots.

### Conflict of Interest Statement

The authors declare that the research was conducted in the absence of any commercial or financial relationships that could be construed as a potential conflict of interest.
